# High-Resolution Monitoring of Antimicrobial Consumption in Vietnamese Small-Scale Chicken Farms Highlights Discrepancies Between Study Metrics

**DOI:** 10.3389/fvets.2019.00174

**Published:** 2019-06-21

**Authors:** Nguyen Van Cuong, Doan Hoang Phu, Nguyen Thi Bich Van, Bao Dinh Truong, Bach Tuan Kiet, Bo Ve Hien, Ho Thi Viet Thu, Marc Choisy, Pawin Padungtod, Guy Thwaites, Juan Carrique-Mas

**Affiliations:** ^1^Oxford University Clinical Research Unit, Ho Chi Minh City, Vietnam; ^2^Faculty of Animal Science and Veterinary Medicine, University of Agriculture and Forestry, Ho Chi Minh City, Vietnam; ^3^Sub-Department of Animal Health and Production, Cao Lãnh, Vietnam; ^4^Department of Veterinary Medicine, University of Can Tho, Can Tho, Vietnam; ^5^MIVEGEC, IRD, CNRS, University of Montpellier, Montpellier, France; ^6^Food and Agriculture Organization of the United Nations, Hanoi, Vietnam; ^7^Nuffield Department of Medicine, Centre for Tropical Medicine and Global Health, Oxford University, Oxford, United Kingdom

**Keywords:** antimicrobial use, chicken, small-scale farms, metrics, quantification, Vietnam

## Abstract

Chicken is, among farmed species, the target of the highest levels of antimicrobial use (AMU). There are considerable knowledge gaps on how and when antimicrobials are used in commercial small-scale chicken farms. These shortcomings arise from cross-sectional study designs and poor record keeping practiced by many such farmers. Furthermore, there is a large diversity of AMU metrics, and it is not clear how these metrics relate to each other. We performed a longitudinal study on a cohort of small-scale chicken farms (*n* = 102) in the Mekong Delta (Vietnam), an area regarded as a hotspot of AMU, from October 2016 to May 2018. We collected data on all medicine products administered to 203 flocks with the following aims: (1) to describe types and quantities of antimicrobial active ingredients (AAIs) used; (2) to describe critical time points of AMU; and (3) to compare AMU using three quantitative metrics: (a) weight of AAIs related to bird weight at the time of treatment (mg/kg at treatment); (b) weight of AAIs related to weight of birds sold (mg/kg sold); and (c) “treatment incidence” (TI), or the number of daily doses per kilogram of live chicken [Vietnamese animal daily dose (ADDvetVN)] per 1,000 days. Antimicrobials contained in commercial feed, administered by injection (*n* = *N* = 6), or antimicrobials for human medicine (*n* = *N* = 16) were excluded. A total of 236 products were identified, containing 42 different AAIs. A total of 76.2% products contained AAIs of “critical importance” according to the World Health Organization (WHO). On average, chickens consumed 791.8 (SEM ±16.7) mg/kg at treatment, 323.4 (SEM ±11.3) mg/kg sold, and the TI was 382.6 (SEM ±5.5) per 1,000 days. AMU was more common early in the production cycle and was highly skewed, with the upper 25% quantile of flocks accounting for 60.7% of total AMU. The observed discrepancies between weight- and dose-based metrics were explained by differences in the strength of AAIs, mortality levels, and the timing of administration. Results suggest that in small-scale chicken production, AMU reduction efforts should preferentially target the early (brooding) period, which is when birds are most likely to be exposed to antimicrobials, whilst restricting access to antimicrobials of critical importance for human medicine.

## Introduction

Antimicrobial resistance (AMR) is a global threat to the health and wealth of nations (1). Antimicrobial usage (AMU) in animal production is regarded as a key driver of AMR in animal populations and a contributor to AMR in humans (2). AMU in animal production has been predicted to increase by 67% from 2010 to 2030 (3), while livestock production may increase by 74% between 1999 and 2030 (4). This increase is mostly driven by increased animal protein consumption in low- and middle-income countries (LMICs).

Chicken meat is the most consumed protein commodity in LMICs because of its comparative advantages. These include the relatively low capital investment and production costs, as well as the lack of religious objections to its consumption (5). In Vietnam, chicken meat currently ranks, after pork, the second most popular type of meat, and by 2020, it is forecast to surpass pork consumption (6).

In 2015, the World Health Organization (WHO) launched its Global Action Plan on AMR, with one of its key objectives being the development and enhancement of monitoring systems for AMU worldwide (7). However, measuring AMU in animal production in LMICs is often challenging due to the large numbers of small-scale farming units, high disease incidence, access of antimicrobials “over the counter,” and generally loose regulatory framework (8). According to the Vietnamese official census (2018), of 245M chickens, only 26.1% corresponded to chickens raised in industrial systems (9), with the remainder corresponding to chickens raised in backyard and small-scale (semi-intensive) commercial farms.

AMU can be measured using a large diversity of metrics (10), and the choice of one metric over the other may lead to inconsistent results (11). Several studies have highlighted a very high level of AMU in Vietnamese chicken production, in terms of both frequency and quantities. A study in 210 poultry farms in northern Vietnam reported the use of 45 different antimicrobial active ingredients (AAIs) (12). A cross-sectional study in the Mekong Delta region indicated that, excluding feed, farmers used approximately 470 mg of AAIs to raise one chicken (13). In terms of treatment intensity, AMU in chicken flocks in a neighboring Mekong Delta province (Tien Giang) was 371 defined daily doses (DDD) per 1,000 chicken-days (14). Factors associated with such a high amount of AMU include ease of access to antimicrobials (i.e., density of veterinary drug shops) and the presence of disease and mortality in flocks, which has been described as very high (15).

However, most published studies in Vietnam (and in other LMICs) on AMU to date are based on cross-sectional study designs (i.e., a one-off visit) focused on the prevalent small-scale farm units. Since many farmers do not keep accurate records on AMU, they are likely to be prone to recall biases (16).

Using longitudinal active surveillance on a large cohort of small-scale commercial chicken flocks, we aimed (1) to describe the types of health-supporting products used, with a focus on antimicrobial active ingredients (AAIs); (2) to describe the critical time points for antimicrobial use (AMU) during the production cycle; and (3) to compare AMU using three common metrics of AMU in chicken production in the Mekong Delta of Vietnam. Detailed information about the types and timing of AMU, as well as its magnitude and the relationship between study metrics, is essential in order to improve the design of national/regional monitoring systems. Furthermore, this should help formulate more targeted campaigns aimed at promoting responsible use of antimicrobials among chicken farmers.

## Materials and Methods

### Farms, Flocks, and Data Collection

The study was conducted from October 2016 to May 2018 during the baseline (observational) phase of a research project (17). Chicken farm owners of two districts (Cao Lanh and Thap Muoi) in the province of Dong Thap (Mekong Delta of Vietnam) were randomly selected from the official farm census held by the veterinary authorities (Sub-Department of Animal Health and Production of Dong Thap, SDAH-DT). These two study districts were chosen based on the availability of qualified veterinary staff to conduct the study. The two chosen districts have, on average, a human population of 331 and 354 chickens per square kilometer (2011); these figures are close to the average for the whole Mekong Delta region (410 humans and 478 chickens per square kilometer) (2011).

Farm owners registered in the census (*n* = 207) were convened and introduced to the project. Farmers intending to raise chickens in flocks of >100 chickens were invited to join the study prospectively as soon as they restocked their follow-on cycle. Project staff provided participating farmers with purposefully designed record books organized by week, where they were requested to record in detail the quantities of all health-supporting products used (including antimicrobial-containing products). Farmers were also asked to keep all packages (bottles, sachets, etc.) of any products purchased/used in their flock in a dedicated container. Study farms were visited four times during each flock production cycle to review the product containers (i.e., active ingredients, function, concentration, and instructions for use) and to verify the collected data. All data (commercial product names and quantities used) were entered into a database using a web-based application. The information collected included number of chickens present in the flock each week and the number of chickens that died over the week. From these data, the flock cycle (cumulative) incidence of mortality was calculated for each production cycle by dividing the total number of birds that died during the period from restocking to sale by the total number of birds restocked for that cycle. A total of 203 flocks that completed at least one entire cycle (from 1-day-old chick until all chicken sold) raised in 102 farms were investigated. Of the 102 farms, 33 (32.3%) completed one cycle, 40 (39.2%) completed two cycles, 19 (18.6%) completed three cycles, 8 (7.8%) completed four cycles, and 2 (19.6%) completed five cycles. Recruited flocks ranged between 100 and 1,530 chickens at restocking. The median flock size at restocking was 300 [Inter-quartile range (IQR) 200–495]. The median duration of one production cycle was 18 [IQR 16–20] weeks, and the median cumulative mortality over the whole production cycle of flocks was 14.1% [IQR 6.8–29.2].

### Description of Health-Supporting Medicinal Products

All health-supporting medicinal products were identified by their composition, and those products containing antimicrobials were singled out. They were described by type (human or veterinary medicine), composition (antimicrobial active ingredient only or mixed with other substances), number of active ingredients, administration route (drinking water, feed, injection), and formulation (powder, liquid). AAIs were classified based on the World Organisation for Animal Health (OIE) list of antimicrobial agents (18).

### Timing of Antimicrobial Usage

The probability of a flock being medicated by age (production week) was calculated by dividing the total number of flocks where at least one antimicrobial-containing product was administered by the total number of flocks observed in the same week. In order to investigate potential seasonal variations in AMU, a Lexis diagram was created, with both the probabilities of AMU by production week and week calendar time plotted. A generalized logistic model was fitted with flock identity as the clustering variable and age and calendar week (sine and cos transformed) as covariates. The timing of AMU was investigated for the 20 most commonly used AAIs. The distribution of times of usage of each AAI from week 1 to week 21 (last week of AMU) was plotted.

### Quantification of Antimicrobial Usage

The total live weight (body mass in kilograms) of chickens present in each flock at each week was calculated from the number of chickens present in the flock and their estimated weight. The latter was based on weekly weightings of 10 randomly-selected chickens from each of 11 representative flocks, from week 1 until week 22 of their production cycle (Supplementary Data 1). The amounts of AAI administered were calculated from farmers' records. The following two weight-based metrics were calculated: (1) weight of active ingredient related to the weight of bird at the time of treatment (mg/kg at treatment) and (2) weight of active antimicrobial active ingredient given over the whole production cycle related to weight of chickens sold (mg/kg sold). This was estimated from the number of chickens present in the flock and their weight at the time of sale. The instructions for mixing the products in water and/or feed (dilution factor) and the estimated daily water and feed consumption were used to estimate for each AAI the daily dose (in mg) associated with treating 1 kg of chicken (ADDvetVN). The weekly water consumption was estimated from the daily intake of a standard meat type pullet at an ambient temperature of 32°C (225 ml per kilogram of live chicken) (19); the weekly feed consumption was estimated from published data related to native Vietnamese layer pullets (i.e., 63.4 g daily per kilogram of live chicken) (20). The expressions used for the calculation of the above metrics are provided in Supplementary Material S1.

The number of ADDvetVN of each AAI administered on any given week to each flock (nADDvetVN) was inferred from the amounts of antimicrobial products consumed. The total nADDvetVN administered was divided by the duration of the cycle (in weeks) and multiplied by 1,000. This “treatment incidence” (TI) can be interpreted as the number of days (per 1,000 days) when one chicken is treated.

For antimicrobial products containing two or four AAIs, the number of doses (nADDvetVN) assigned to each AAI contained in the product was calculated as the total number of doses associated with the product divided by two or four, respectively. Products administered through the parenteral route (injection) and human medicines (tablets) were excluded, since the number of chickens receiving injection was not recorded, and guidelines for preparation of human medicines were not available. In addition, antimicrobials contained in purchased commercial feeds were not included in the analyses since they contained ambiguous formulations. Quantitative AMU metrics at the flock level were compared using Pearson's correlation coefficient (PCC). We calculated the mean and coefficient of variation of ADDvetVN values corresponding to AAIs present in Vietnamese antimicrobials and compared them with the DDDvet values defined for poultry by the European Medicines Agency (21).

## Results

### Health-Supporting Products

A total of 619 different health-supporting products were identified among the 203 flocks investigated, of which 236 (38.1%) contained antimicrobials (Table 1). The most common non-antimicrobial health-supporting products (*n* = 383) consisted (in decreasing order) of vitamins/minerals (21.5%), digestive enzymes (8.1%), vaccines (3.7%), coccidiostats (3.6%), electrolytes (3.6%), anthelminthics (2.9%), and interferon/immunoglobulins (0.5%). Of the 112 “other” categories of product, most (~80%) were anti-inflammatory/anti-pyretic products (i.e., paracetamol, prednisolone).

**Table 1:** Summary of health-supporting products used by study flocks.

**Type of product**	**No. of products (*n* = 619) (%)**	**Farms (*n* = 102) (%)**	**Flocks (*n* = 203) (%)**	**Weeks (*n* = 3,663) (%)**
Antimicrobial-containing	236 (38.1)	100 (98.0)	192 (94.5)	933 (25.5)
Non-antimicrobial	383 (61.9)	102 (100)	202 (99.5)	2,128 (63.3)
Vitamins/minerals	133 (21.5)	99 (97.1)	189 (93.6)	1,428 (67.1)
Probiotics	50 (8.1)	86 (84.3)	157 (77.7)	942 (44.3)
Vaccines	23 (3.7)	102 (100)	203 (100)	784 (29.4)
Coccidiostats	22 (3.6)	76 (74.5)	137 (67.8)	304 (14.3)
Electrolytes	22 (3.6)	63 (61.8)	100 (49.5)	299 (14.1)
Anthelminthics	18 (2.9)	49 (48)	71 (35.1)	96 (4.5)
Interferon/immunoglobulins	3 (0.5)	88 (86.3)	144 (71.3)	293 (13.8)
Other (unclassified)	112 (18.1)	81 (79.4)	139 (68.8)	517 (24.3)

Of the 236 antimicrobial-containing products, 176 (74.5%) contained only AAIs (apart from excipient), whereas 25.5% contained AAIs mixed with other substances (i.e., vitamins, mineral, electrolytes, anti-inflammatory, and anti-pyretic substances). A total of 141 (59.7%) products contained two AAIs, and 1 (0.4%) contained four AAIs. Overwhelmingly, 227 products (96.2%) were intended for oral administration and 215 products (91.1%) were intended for powder-based formulations (Table 2). A total of 16 human medicine products were used by 4.4% of the study flocks. Antimicrobials were used in 25.5% observation weeks (*n* = 3,663).

**Table 2:** Description of antimicrobial-containing products administered to 203 chicken flocks.

**Category**	**Sub-category**	**Products (*n* = 236) (%)**	**Farms (*n* = 102) (%)**	**Flocks (*n* = 203) (%)**	**Week (*n* = 3,663) (%)**
Type of product	Animal medicine	220 (93.2)	100 (98.0)	191 (94.1)	697 (19.0)
	Human medicine	16 (6.8)	6 (5.9)	9 (4.4)	32 (0.9)
Composition	AAI only	176 (74.6)	92 (90.3)	169 (83.2)	629 (16.9)
	AAIs mixed with other substances	60 (25.4)	87 (85.3)	162 (79.8)	448 (12.2)
No. of AAIs per product	One	94 (39.9)	78 (76.5)	135 (66.5)	359 (9.8)
	Two	141 (59.7)	100 (98.0)	190 (93.6)	697 (19.0)
	Four	1 (0.4)	1 (1.0)	1 (0.5)	3 (0.1)
Administration route	Oral	227 (96.2)	100 (98)	192 (95.5)	928 (25.3)
	Oral—water	209 (88.9)	98 (96.1)	191 (94.1)	860 (23.7)
	Oral—feed	21 (8.9)	31 (29.4)	35 (17.2)	190 (5.2)
	Injection	6 (2.5)	13 (12.7)	14 (6.9)	19 (0.5)
Type of formulation	Powder	215 (91.1)	100 (98.0)	191 (94.1)	889 (24.3)
	Liquid	21 (8.9)	36 (35.3)	43 (21.2)	73 (1.9)

*AAI, antimicrobial active ingredients*.

### Description of Antimicrobial Active Ingredients

A total of 42 different AAIs belonging to 13 classes were identified (Table 3). A total of 180 (76.2%) products contained antimicrobials of critical importance according to the WHO (22). Of those, 132 (55.9%) products contained AAIs of critical importance (“highest priority”) and 91 (38.5%) products contained critically important (“high priority”) antimicrobials. The most common AAI used were colistin (25.8% products, 83.7% flocks), followed by oxytetracycline (15.7%; 76.4%), tylosin (13.6%; 36.9%), doxycycline (11%; 30%), and amoxicillin (10.2%, 24.6%) (Table 3). Antimicrobials for human use consisted of tablets containing amoxicillin and tetracycline AAI (three products each); ampicillin, chloramphenicol, ciprofloxacin, and sulfaguanidine (two products each); and cefotaxime (one product). Supplementary Material S2 includes the list of all AAIs contained in all antimicrobial products investigated.

**Table 3:** AAIs administered to study flocks.

**Antimicrobial class**	**AAI**	**Products (*n* = 236) (%)**	**Farms (*n* = 102) (%)**	**Flocks (*n* = 203) (%)**	**Weeks (*n* = 3,663) (%)**
Aminoglycosides^*^	Neomycin	17 (7.2)	33 (32.4)	43 (21.2)	85 (3.1)
	Gentamicin	15 (6.4)	41 (40.2)	60 (29.6)	87 (3.2)
	Streptomycin	8 (3.4)	30 (29.4)	41 (20.2)	79 (2.9)
	Spectinomycin	7 (3)	10 (9.8)	12 (5.9)	18 (0.6)
	Apramycin	1 (0.4)	3 (2.9)	3 (1.5)	3 (0.1)
	*Any aminoglycoside*	50 (21.2)	69 (67.6)	115 (56.7)	259 (9.7)
Amphenicols	Florfenicol	13 (5.5)	24 (23.5)	27 (13.3)	40 (1.5)
	Thiamphenicol	3 (1.3)	20 (19.6)	27 (13.3)	36 (1.3)
	Chloramphenicol	2 (0.8)	2 (2.0)	5 (2.5)	15 (0.5)
	*Any amphenicol*	18 (7.6)	40 (39.2)	53 (26.1)	90 (3.4)
1st- and 2nd-gen. cephalosporins	Cefadroxil	1 (0.4)	1 (1.0)	1 (0.5)	2 (0.1)
	Cefotaxime	1 (0.4)	1 (1.0)	1 (0.5)	1 (0.0)
	Cefalexin	1 (0.4)	1 (1.0)	1 (0.5)	1 (0.0)
	*Any 1st and 2nd gen. cephalosporin*	2 (0.8)	2 (2.0)	2 (1.0)	4 (0.2)
Diaminopyrimidines	Trimethoprim	17 (7.2)	31 (30.4)	39 (19.2)	72 (2.7)
Lincosamides	Lincomycin	13 (5.5)	16 (15.7)	21 (10.3)	32 (1.2)
Macrolides^**^	Tylosin	32 (13.6)	48 (47.1)	75 (36.9)	160 (6.0)
	Tilmicosin	7 (3)	20 (19.6)	24 (11.8)	37 (1.3)
	Erythromycin	6 (2.5)	16 (15.7)	18 (8.9)	27 (1.0)
	Spiramycin	6 (2.5)	11 (10.8)	12 (5.9)	15 (0.5)
	Kitasamycin	1 (0.4)	1 (1.0)	1 (0.5)	1 (0.0)
	Josamycin	1 (0.4)	2 (2.0)	2 (1.0)	4 (0.1)
	*Any macrolide*	51 (21.6)	57 (55.9)	91 (44.8)	227 (8.5)
Penicillins^*^	Amoxicillin	24 (10.2)	43 (42.2)	50 (24.6)	87 (3.2)
	Ampicillin	17 (7.2)	27 (26.5)	38 (18.7)	78 (2.9)
	*Any penicillin*	41 (17.4)	56 (54.9)	91 (44.8)	164 (6.2)
Pleuromutilins	Tiamulin	1 (0.4)	1 (1)	1 (0.5)	1 (0.0)
Polypeptides^**^	Colistin	61 (25.8)	94 (92.2)	170 (83.7)	413 (15.5)
	Enramycin	1 (0.4)	1 (1.0)	1 (0.5)	1 (0.0)
	*Any polypeptide*	61 (25.8)	94 (92.2)	170 (83.7)	414 (15.5)
Quinolones/fluoroquinolones^**^	Enrofloxacin	13 (5.5)	32 (31.4)	45 (22.2)	76 (2.8)
	Flumequine	9 (3.8)	12 (11.8)	16 (7.9)	27 (1.0)
	Norfloxacin	2 (0.8)	7 (6.9)	9 (4.4)	13 (0.4)
	Ciprofloxacin	2 (0.8)	2 (2.0)	3 (1.5)	5 (0.2)
	Marbofloxalin	1 (0.4)	1 (1.0)	1 (0.5)	1 (0.0)
	*Any quinolone*	27 (11.4)	42 (41.2)	66 (33.5)	122 (4.6)
Sulfonamides	Sulphamethoxazole	7 (3.0)	26 (25.5)	34 (16.7)	68 (2.5)
	Sulfadimidine	6 (2.5)	8 (7.8)	9 (4.4)	11 (0.4)
	Sulfadimethoxine	6 (2.5)	14 (13.7)	16 (7.9)	21 (0.8)
	Sulfaguanidin	2 (0.8)	2 (2.0)	4 (2.0)	11 (0.4)
	Sulfadiazine	2 (0.8)	2 (2.0)	2 (1.0)	4 (0.1)
	Sulfamethoxypyridazine	1 (0.4)	2 (2.0)	2 (1.0)	4 (0.1)
	Sulfachloropyridazine	1 (0.4)	1 (1.0)	1 (0.5)	1 (0.0)
	Sulfamethazine	1 (0.4)	1 (1.0)	1 (0.5)	1 (0.0)
	Sulfathiazole	1 (0.4)	1 (1.0)	1 (0.5)	1 (0.0)
	*Any sulfonamide*	25 (10.6)	45 (44.1)	60 (29.6)	118 (4.4)
Tetracyclines	Oxytetracycline	37 (15.7)	87 (85.3)	155 (76.4)	332 (12.4)
	Doxycycline	26 (11.0)	42 (41.2)	61 (30.0)	129 (4.8)
	Tetracycline	6 (2.5)	7 (6.9)	10 (4.9)	28 (1.0)
	*Any tetracycline*	69 (29.2)	93 (91.2)	173 (85.2)	474 (17.8)
Unclassified	Methenamine	1 (0.4)	15 (14.7)	23 (11.3)	31 (1.1)

Critically important antimicrobial classes according to the World Health Organization (WHO) are highlighted:

* High priority,

** *Highest priority*.

### Antimicrobial Use by Week

**Figure 1:**
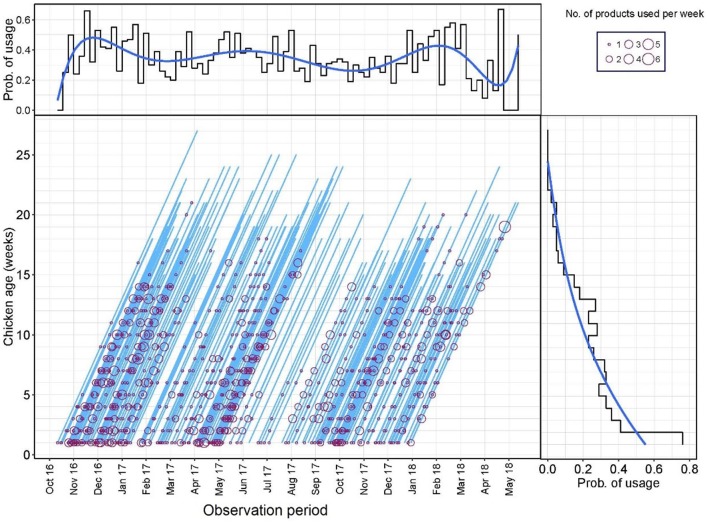
Lexis diagram and probability of antimicrobial use (AMU) (Yes/No) by production week and calendar week during the study period.

A Lexis diagram displaying the probability of AMU of flocks by production age and calendar time (weeks) is shown in Figure 1. The probability of AMU decreased with the age of the flock (from 0.76 in week 1, 0.41 in week 2, and 0.02 in week 21). From the Lexis graph, there was an indication of increased AMU during certain calendar periods (peaks in December 2016, June 2017, and February 2018). However, when both variables were fit into the same logistic model with the probability of AMU as an outcome, only the age of the flock (weeks) was significant (data not shown). A median of 5.0 [IQR 2.25–10.0] products and 6.0 [IQR 3.0–10.0] AAIs were used in each flock cycle.

### Timing of Antimicrobial Use

**Figure 2:**
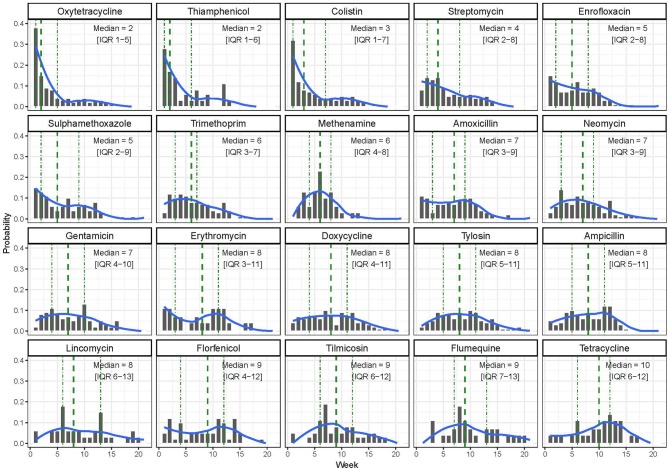
Distribution of the timing of use of the 20 most common antimicrobial active ingredients (AAIs) by week among study flocks.

In terms of timing of use, the AAIs used earlier in the production cycle were oxytetracycline [median timing of use, 2 weeks (IQR 1–5)], thiamphenicol [median 2.0 (IQR 1.0–6.0)], and colistin [median 3 (IQR 1.0–7.0)]. Tilmicosin [median 9 (IQR 6.0–12.0)], flumequine [median 9.0 (IQR 7.0–13.0)], and tetracycline [median 10.0 (IQR 6.0–12.0)] were the three AAIs that were administered latest to study flocks (Figure 2).

### Quantification of Antimicrobial Use

**Figure 3:**
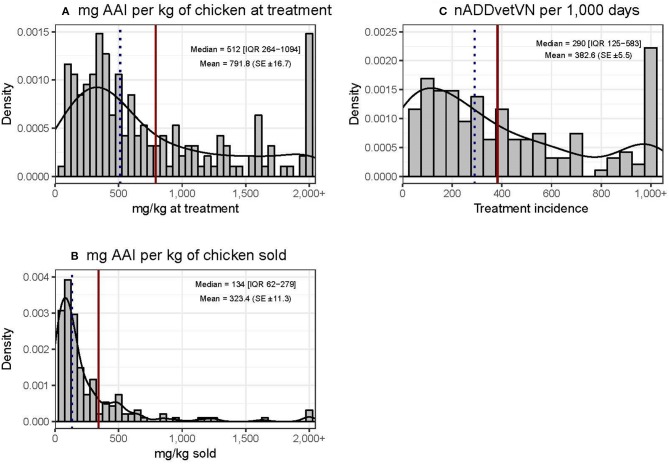
Distribution of quantitative metrics of AMU in study flocks. Dotted black line: median. Solid red line: mean.

Chicken flocks were administered a mean of 791.8 (±16.7) mg AAI per kilogram of chicken at treatment time [median 512 mg (IQR 264–1,094)] and 323.4 (±11.3) mg per kilogram of chicken sold [median 134 mg (IQR 62–279)]. The mean TI was 382.6 (±5.5) ADDs per 1,000 days [median 290 (IQR 125–583) per 1,000 days] (Figure 3). These calculations excluded AAIs contained in commercial feed, injectables, or human medicine antimicrobials. The data were quite skewed in all three metrics, with the mean being always greater than the median value. In terms of mg/kg at treatment, the upper 25% quantile of flocks accounted for 60.7% of total use. In addition, 23 (12.0%) flocks used more than 1,000 doses per 1,000 chicken days. For the “mg/kg sold” metric calculation, 9/203 (4.4%) flocks were excluded, since they experienced 100% mortality and therefore no live chickens were sold from such flocks.

Tetracyclines were the most used antimicrobial class reflected in both metrics: 285.1 mg/kg at treatment (SEM ±23.4) and a TI of 150.9 (±9.3) per 1,000 days. In terms of mg/kg at treatment, the highest magnitude of AMU corresponded to oxytetracycline 231.5 mg (29.2%), methenamine 105.8 mg (13.2%), and amoxicillin 48.7 mg (6.2%); in contrast, the highest TI corresponded to colistin 145.5, oxytetracycline 141.8, and enrofloxacin 16.1 (Table 4).

**Table 4:** Amounts of AAIs used through the oral route in study flocks.

		**Mean AMU by flock (±SEM)**		
**Antimicrobial class**	**AAI**	**mg/kg at treatment**	**mg/kg sold**	**Treatment incidence**
Aminoglycosides	Neomycin	38.0 (±16.4)	14.7 (±5.9)	4.4 (±1.1)
	Gentamicin	12.5 (±3.2)	6.3 (±3.5)	2.1 (±0.4)
	Streptomycin	22.5 (±10.5)	14.3 (±16)	6.0 (±1.3)
	Spectinomycin	2.2 (±3)	0.6 (±0.7)	1.0 (±1.0)
	Apramycin	0.5 (±1.1)	1.2 (±7.2)	<0.1 (±nc)
	Josamycin	0.9 (±3.2)	7.5 (±68)	<0.1 (±nc)
	Total aminoglycosides	75.7 (±5.9)	37.5 (±24.2)	13.5 (±2.7)
Amphenicols	Florfenicol	7.3 (±3.7)	9.4 (±12.1)	1.9 (±0.8)
	Thiamphenicol	26.2 (±12.5)	4.4 (±3.7)	3.1 (±0.6)
	Chloramphenicol	nc	nc	nc
	Total amphenicols	33.5 (±6.6)	13.8 (±1.2)	5.0 (±1.6)
1st and 2nd gen. cephalosporins	Cefadroxil	0.5 (±nc)	<0.1 (±nc)	<0.1 (±nc)
	Cefotaxime	nc	nc	nc
	Cefalexin	<0.1 (±nc)	<0.1 (±nc)	<0.1 (±nc)
	Total	0.5 (±nc)	<0.1 (±nc)	<0.1 (±nc)
Diaminopyrimidines	Trimethoprim	25.7 (±nc)	11.7 (±nc)	4.3 (±nc)
Lincosamides	Lincomycin	3.2 (±nc)	2.3 (±nc)	1.4 (±nc)
Macrolides	Tylosin	34.8 (±8.5)	27.7 (±17.3)	6.5 (±1.2)
	Tilmicosin	25.9 (±19.2)	20.9 (±25.4)	7.8 (±4.6)
	Erythromycin	12.2 (±16.1)	5.7 (±12.3)	3.8 (±2.9)
	Spiramycin	1.5 (±1.4)	0.2 (±0.5)	1.1 (±0.5)
	Kitasamycin	<0.1 (±nc)	0.4 (±nc)	<0.1 (±nc)
	Josamycin	0.9 (±3.2)	7.5 (±68)	<0.1 (±nc)
	Total	75.3 (±7.9)	62.0 (±10.4)	19.2 (±7.5)
Penicillins	Amoxicillin	48.7 (±24.7)	25.8 (±28.7)	14.4 (±3.4)
	Ampicillin	11.1 (±6.1)	5.5 (±4)	1.5 (±0.8)
	Total	59.8 (±13.2)	31.3 (±17.5)	15.9 (±7.5)
Pleuromutilins	Tiamulin	<0.1 (±nc)	<0.1 (±nc)	<0.1 (±nc)
Polypeptides	Colistin	41.6 (±5.7)	8.8 (±1.6)	145.8 (±4.6)
	Enramycin	<0.1 (±nc)	<0.1 (±nc)	<0.1 (±nc)
	Total	41.6 (±3.5)	8.8 (±0.9)	145.8 (±5.9)
Quinolones/Fluoroquinolones	Enrofloxacin	24.1 (±8.4)	7.4 (±4.6)	16.1 (±2.6)
	Flumequine	5.4 (±3.2)	3.4 (±2)	0.6 (±0.2)
	Norfloxacin	6.4 (±6.5)	2.4 (±3.5)	1.1 (±0.8)
	Ciprofloxacin	nc	nc	nc
	Marbofloxalin	nc	nc	nc
	Total	35.9 (±5.6)	13.2 (±4.8)	17.8 (±7.8)
Sulfonamides	Sulphamethoxazole	30.2 (±1.2)	11.7 (±15.1)	3.6 (±0.6)
	Sulfadimidine	4.1 (±4.8)	2.3 (±2.5)	0.1 (±nc)
	Sulfadimethoxine	13.5 (±27.7)	2.4 (±2)	1.9 (±1.4)
	Sulfaguanidin	nc	nc	nc
	Sulfadiazine	2.4 (±10)	0.7 (±4.8)	0.2 (±0.3)
	Sulfamethoxypyridazine	0.5 (±2)	0.3 (±0.8)	<0.1 (±nc)
	Sulfachloropyridazine	<0.1 (±nc)	<0.1 (±nc)	<0.1 (±nc)
	Sulfamethazine	0.7 (±nc)	<0.1 (±nc)	1.0 (±nc)
	Sulfathiazole	<0.1 (±nc)	<0.1 (±nc)	<0.1 (±nc)
	Total	51.4 (±9.5)	17.4 (±5.1)	4.9 ±1.4)
Tetracyclines	Oxytetracycline	231.5 (±21.0)	43.7 (±9.8)	141.8 (±4.6)
	Doxycycline	42.6 (±13.3)	14.0 (±3.4)	7.5 (±1.2)
	Tetracycline	7.4 (±46.8)	7.9 (±52.5)	1.6 (±4.0)
	Total	285.1 (±23.4)	65.6 (±27.9)	150.9 (±9.3)
Unclassified	Methenamine	105.8 (±nc)	58.0 (±nc)	1.1 (±nc)
Total		791.8 (±16.7)	323.4 (±11.3)	382.6 (±5.5)

*nc, not calculated*.

### Correlation Between Antimicrobial Use Metrics

**Figure 4:**
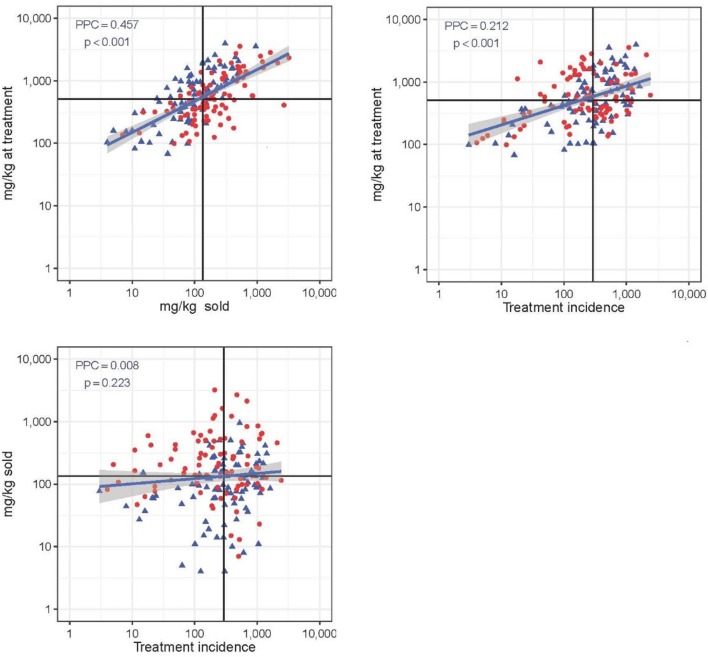
Correlation between three quantitative AMU metrics (“mg/kg at treatment,” “mg/kg sold,” and “treatment incidence”). Solid black lines represent the median value of each metric. PCC is Pearsons's correlation coefficient. Red dot: flock with high (≥14.1%) mortality; blue dot: flock with low (<14.4%) mortality. Note the log 10-transformed scale for easier visualization.

Figure 4 shows the three correlation plots between each pair of the three AMU metrics used. Correlation was highest between “mg/kg sold” and “mg/kg at treatment” (PCC = 0.457; *p* < 0.001) (moderate positive relationship). The metric “mg/kg at treatment” was weakly correlated with “treatment incidence” (PCC = 0.212; *p* < 0.001). There was no correlation between TI and mg/kg sold metric (PCC = 0.008; *p* = 0.223). The proportion of flocks with high mortality (≥14.1%) was significantly greater among flocks with higher than average AMU expressed with the mg/kg sold metric (0.64 vs. 0.34, χ^2^ = 15.52; *p* < 0.001). In the case of the other two metrics, there were no significant differences in mortality between high and low AMU users (both *p* > 0.407).

### Vietnamese Animal Daily Dose for Chicken Production

The mean ADDvetVN corresponding to each of 37 AAIs was calculated from 223 different veterinary medicine products (Supplementary Material S3). ADDvetVN values ranged from 4.4 mg (sulfamethazine) to 320.6 mg (methenamine). However, most of the values were lower than 50 mg (35/38 AAI). A very high coefficient of variation (>100%) was also observed in several AAIs such as colistin, gentamicin, doxycycline, trimethoprim, tylosin, neomycin, spectinomycin, sulfadimidine, and florfenicol. There were 27 AAIs with data on DDDvet for poultry available in the European Union (EU). Of those, 14/27 antimicrobials from Vietnamese products had lower ADDs, while 13/27 had higher ADDs. Notably, the values of several DDDvet from the EU (i.e., spectinomycin, tylosin, ampicillin, and spiramycin) were four to five times higher than ADDvetVN.

### Antimicrobial Use by Antimicrobial Active Ingredients

**Figure 5:**
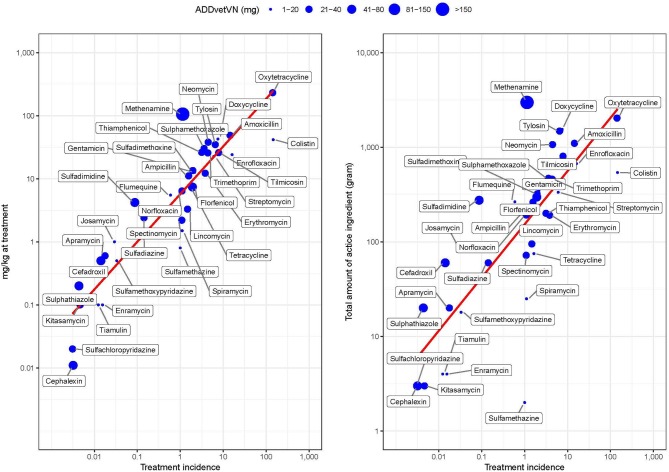
Relationship between study metrics by AAI in study flocks. The size of the dot is proportional to the magnitude of the Vietnamese animal daily dose (ADDvetVN) for each AAI (in mg). Note the log 10-transformed scale for easier visualization.

Figure 5 shows the correlation between TI and weight-based metrics (mg/kg at treatment and total weight of antimicrobials ignoring population treated) by AAI (Supplementary Material S3). The two metrics were moderately correlated (PPC > >0.480, *p* < 0.001 in both cases). However, the greater deviation from perfect correlation was observed for those AAIs with very low (i.e., colistin) or very high (i.e., methenamine) ADDvetVN values (5.2 and 320.6 mg/kg chicken, respectively). Comparing antimicrobials with similar TI, such as methenamine and spectinomycin (i.e., both ~1 ADD per 1,000 chicken-days), given that the former has a much higher ADDvetVN value (320.6 mg/kg) than the latter (33 mg/kg), this results in quantitatively larger estimates for methenamine in terms of “total amounts (grams) of active ingredient” (Figure 5, right).

## Discussion

Our study deliberately focused on small-scale commercial farming systems. In doing so, we excluded both larger industrial (broiler) and backyard production systems. The small-scale commercial chicken sector represented here, alongside industrial broiler production, is increasingly important in Vietnam: from 2011 to 2016 the number of registered units raising more than 100 chickens has experienced a 41.5% increase (23).

Using three different metrics, this study provided an accurate characterization of AMU in small-scale chicken flocks in the Mekong Delta of Vietnam, an area regarded as a hotspot of AMU. AMU levels were 791.8 (SEM ±16.7) mg of AAI per kilogram at treatment and 323.4 (SEM ±11.3) mg per kilogram sold. In terms of TI, chicken flocks were treated on average 382.6 days (SEM ±5.5) per 1,000 days. These results excluded antimicrobials included in purchased commercial feed formulations and a few antimicrobial products that were administered through the injectable route or human medicine antimicrobials products. In Vietnam, antimicrobials included in commercial feed have been quantified to be in the order of 77.4 mg per kilogram of live chicken raised in a previous study. In terms of TI, chickens in our study consumed three times more than global average levels (estimated in 138.0 doses per 1,000 chicken-days) (10).

It is particularly concerning that around three quarters (76.2%) of the products examined contained AAIs of “critical importance,” and over half (55.9%) contained at least one AAI of critical importance (highest priority) according to the WHO (i.e., colistin, quinolones, and macrolides). The magnitude of colistin use is of particular concern, since this is one of the antimicrobials of last resort for hospital-acquired infections in humans (24). Colistin was found either alone or in combination with other antimicrobials such as oxytetracycline, ampicillin, neomycin, tylosin, enrofloxacin, etc. A possible reason for its popularity is its low cost, since it is an older-generation antimicrobial. Most (~60%) antimicrobial-containing products were formulated with two AAIs. This scenario is different from European countries, where one active ingredient is allowed, except for a few drugs that are always formulated as combination (i.e., trimethoprim and sulphonamides) (21). In a small percentage of flocks (4%), we found that farmers had used chloramphenicol, an antimicrobial that has been banned for almost two decades in the country (25). In 2% of farms, ciprofloxacin (also banned for use in animal production) had also been used. We found a large number of farms that administered more doses than those technically necessary over the life of the flock. We believe that this is a reflection of errors in the preparation resulting in excessive concentration of the AAI during the early phases, since the costs of administering antimicrobials in small birds is relatively lower.

Results from this study highlight significant discrepancies between metrics. Relating AMU to chicken weight at treatment results in estimates of a magnitude two to three times higher than relating AMU to chicken weight at the end of the production cycle. The “mg/kg at treatment” metric was largely influenced by the timing of AMU, with higher values resulting from administration of the product early in the production cycle (i.e., brooding), therefore resulting in larger estimates. The “mg/kg at treatment” use is expected to always be higher than “mg/kg sold,” since the weight at the end of production is typically the highest. This latter metric was, however, largely affected by mortality, with flocks experiencing high mortality having considerably higher AMU estimates due to the smaller denominator in such flocks. If national estimates of AMU were to be calculated from production data, it is therefore essential to factor in the high levels of mortality that are typical of each production system. The “treatment incidence” metric is the most balanced overall metric, since it incorporates the variability associated with the variable strength of the AAIs administered. However, a challenge associated with the latter is the definition of a “daily dose,” given that most antimicrobial products included guidelines for both prophylactic and therapeutic use, and information on the actual preparation procedures used by the farmer (dilution factor) was not collected. Indications for prophylactic use involve mixing the product with approximately half the strength of indication for therapeutic use. In addition, most products contain two AAIs, and each AAI amounted to half a theoretical daily dose in the overall calculation. The major discrepancies observed between weight-based and dose-based metrics can be explained because of differences in strength of different AAIs, timing of use, and variable mortality. In situations where AAIs characterized by large technical units are used, calculations using weight-based metrics will result in the overestimation of results using weight-based metrics over treatment incidence metrics.

We report differences in the timing of usage of different antimicrobials. Some antimicrobials, such as tetracycline and tilmicosin, have withdrawal times of over 1 week (26), and in several cases were administered late in the production cycle. This probably explains the high rate of detection of macrolide and tetracycline residues (10.3% each) in chicken meat samples purchased from the study area (27).

The study highlighted a huge diversity of AAIs used by small-scale chicken farmers. In Vietnam, about 10,000 products are currently licensed for veterinary use (28, 29), and ~50% contain AAIs (author's observation). We established the Vietnamese “animal daily dose” for antimicrobials used in chicken production (ADDvetVN). Athough our calculations of ADDvetVN were based on the indication displayed in the label for therapeutic purpose, most values were still lower than the DDDvet from the European Union, and for several AAIs (i.e., spiramycin, ampicillin) they were four to five times lower. In addition, many products included a recommendationa for prophylactic use, where the product is diluted by a factor of two, and the AAI is therefore administered at an even lower concentration. This is a concern, since such low doses may contribute to increased generation of AMR (30).

We are confident that farmers did provide an honest record of all antimicrobial products used and that the data collected in our study accurately represent AMU in these small-scale farming systems. This was possible since project staff were not perceived to judge farmers' practices negatively. However, obtaining longitudinal high-resolution data required several visits during the production cycle, and a considerable degree of both farmer and research staff commitment. Therefore, these types of studies may not be feasible at a large (i.e., national surveillance) scale, unless considerable resources are dedicated. We understand that the small-scale sector is the target of the largest quantities of AMU in Vietnam, and most of this use is for prophylactic purposes (15). This category of farmers should be the focus of policy makers to reduce excessive AMU in animal production. In Vietnam, most antimicrobials used in animal production are procured by farmers in licensed veterinary pharmacies. Because of this, we believe that setting up monitoring systems at these retail points, coupled with detailed animal production statistics (to be collected at local level), would represent a much more cost-effective surveillance system for AMU compared with conducting farm surveys.

Results highlight the need for training chicken farmers to improve their awareness on AMR while discouraging prophylactic use of antimicrobials, particularly during the brooding period. Such training should emphasize the need to improve day-old chick quality and farming practices (biosecurity, cleaning and disinfection, brooding management, and vaccination). Furthermore, in view of the high usage levels of AMU of critical importance (high priority), we recommend authorities to introduce phased restrictions, starting with those AAIs belonging to the highest priority group.

## Data Availability

The raw data supporting the conclusions of this manuscript will be made available by the authors, without undue reservation, to any qualified researcher.

## Ethics Statement

The ViParc project has been granted ethics approval by the Oxford Tropical Research Ethics Committee (OXTREC) (Minimal Risk) (Ref. 5121/16).

## Author Contributions

JC-M, PP, and NC conceived the study. NC, BK, BH, HT, and NV developed data collection methods and carried out field visits. NC, NV, MC, BT, and DP contributed to data analyses. NC, GT, MC, and JC-M contributed to manuscript writing-up and editing.

### Conflict of Interest Statement

The authors declare that the research was conducted in the absence of any commercial or financial relationships that could be construed as a potential conflict of interest.
