# Supplementary material for: High-Resolution Monitoring of Antimicrobial Consumption in Vietnamese Small-Scale Chicken Farms Highlights Discrepancies Between Study Metrics
Source: Front Vet Sci. 2019 Jun 21;6:174. doi: 10.3389/fvets.2019.00174 (PMC6598194; doi:10.3389/fvets.2019.00174)
Supplement: Supplementary file 1 [file Table_1.DOCX]

**Supplementary Material S1.** Expressions used to calculate AMU in study flocks.

**Expression 1**: Weight of active ingredient related to the weight of bird at the time of treatment (mg per kg at treatment) for all weeks (*n*).

mg/kg at treatment = $\sum_{k=1}^{n} \frac{AAI used \left( mg \right) in week k}{Standard weight of the flock \left( kg \right) at week k}$

**Expression 2:** Weight of active antimicrobial active ingredient related to weight of chickens sold at the end of the production cycle (mg/kg sold).

mg/kg sold = $\frac{\sum_{k=1}^{n} AAI used \left( mg \right) in week k}{Standard weight of the flock \left( kg \right) at week n}$

**Expression 3:** Calculation of ADDvetVN for each AAI present in product (for products to be mixed with water).

ADDvetVN = $\frac{mg of AAI \left( per 100g or product \right)*Dilution factor\left( \frac{l}{mg} \right)}{0.225 l}$

Where 0.225 is the daily consumption of water and 0.063 is the daily consumption of feed of a 1 kg chicken. The dilution factor is specific for each product

**Expression 4:** Calculation of ADD_kg_vetVN for each AAI present in product (products to be mixed with feed).

ADDvetVN = $\frac{mg of AAI \left( per 100g or product \right)*Dilution factor\left( \frac{kg}{mg} \right)}{0.063 kg}$

Where 0.225 is the daily consumption of water and 0.063 is the daily consumption of feed of a 1 kg chicken. The dilution factor is specific for each product.
