# Supplementary material for: High-Resolution Monitoring of Antimicrobial Consumption in Vietnamese Small-Scale Chicken Farms Highlights Discrepancies Between Study Metrics
Source: Front Vet Sci. 2019 Jun 21;6:174. doi: 10.3389/fvets.2019.00174 (PMC6598194; doi:10.3389/fvets.2019.00174)
Supplement: Supplementary file 3 [file Table_3.docx]

**Supplementary Material S3.** Summary of ADDvetVN (mg/kg of live chicken) for 37 AAI from 223 antimicrobial products (AAI included in human medicine products only and administered by the injection route were excluded).

| Antimicrobial active ingredient (AAI) | Products with 1 AAI | | Products with 2 AAIs | | Products with 4 AAIs | | All products | | European Poultry DDDvet |
| --- | --- | --- | --- | --- | --- | --- | --- | --- | --- |
|  | No. products | Mean (±CV) ADDvetVN | No. product | Mean (±CV) (ADDvetVN | No. products | Mean (±CV) ADDvetVN | No. products | Mean (±CV) ADDvetVN |  |
| Sulfamethazine | - | - | 1 | 4.4 | - | - | 1 | 4.4 | - |
| Enramycin | - | - | 1 | 4.5 | - | - | 1 | 4.5 | - |
| Colistin | 2 | 8.5 (±4.7) | 59 | 5.1 (±125.5) | - | - | 61 | 5.2 (±121.1) | 5.1 |
| Tiamulin | - | - | 1 | 6 | - | - | 1 | 6.0 | 23 |
| Enrofloxacin | 12 | 10.2 (±29.4) | 1 | 2.3 | - | - | 13 | 9.6 (±37.5) | 10 |
| Sulfamethoxypyridazine | - | - | 1 | 10.1 | - | - | 1 | 10.1 | 23^*^ |
| Spiramycin | - | - | 6 | 12.6 (±97.6) | - | - | 6 | 12.6 (±97.6) | 73 |
| Gentamicin | - | - | 15 | 13.1 (±109.9) | - | - | 15 | 13.1 (±109.9) | - |
| Streptomycin | 1 | 13.5 | 7 | 16.3 (±44.1) | - | - | 8 | 16.0 (±42.5) | - |
| Tetracycline | 3 | - | 3 | 16.7 (±32.3) | - | - | 6 | 16.7 (±32.3) | 71 |
| Flumequine | 9 | 17.3 (±49.1) | - | - | - | - | 9 | 17.3 (±49.1) | 14 |
| Doxycycline | 5 | 20.7 (±71.5) | 21 | 18.3 (±109.8) | - | - | 26 | 18.7 (±101.1) | 15 |
| Josamycin | - | - | 1 | 19.8 | - | - | 1 | 19.8 | - |
| Trimethoprim | 2 | 2.9 (±1.0) | 14 | 25.2 (±205.1) | 1 | 13.5 | 17 | 21.9 (±215.5) | 6.4^**^ |
| Ampicillin | 1 | 4.0 | 14 | 23.8 (±66.4) | - | - | 17 | 22.4 (±71.4) | 108 |
| Sulfachloropyridazine | - | - | 1 | 22.5 | - | - | 1 | 22.5 | 30^*^ |
| Amoxicillin | 7 | 27.6 (±47.1) | 14 | 20.5 (±64.4) | - | - | 24 | 22.9 (±58.0) | 16 |
| Lincomycin | 3 | 35.9 (±154.8) | 9 | 19.6 (±82.6) | - | - | 13 | 23.7 (±119.8) | 8.6 |

Table S3 (cont): Estimation of ADDvetVN (mg/kg of live chicken) for 37 active ingredients from 223 products (AAI included in human medicine products only and administered by the injection route were excluded).

| Antimicrobial active ingredient (AAI) | Products with 1 AAI | | Products with 2 AAIs | | Products with 4 AAIs | | All products | | European Poultry DDDvet |
| --- | --- | --- | --- | --- | --- | --- | --- | --- | --- |
|  | No. products | Mean (±CV) ADDvetVN | No. products | Mean (±CV) ADDvetVN | No. products | Mean (±CV) ADDvetVN | No. products | Mean (±CV) ADDvetVN |  |
| Thiamphenicol | - | - | 3 | 24.9 (±67.0) | - | - | 3 | 24.9 (±67.0) | 55 |
| Tylosin | 1 | 29.2 | 30 | 25.2 (±108.3) | 1 | 33.1 | 32 | 25.5 (±103.5) | 81 |
| Sulfadiazine | - | - | 1 | 18 | 1 | 33.8 | 2 | 25.9 (±43.2) | 34 |
| Erythromycin | 2 | 33.3 (±45.9) | 4 | 23.6 (±35.1) | - | - | 6 | 26.8 (±39.5) | 20 |
| Oxytetracycline | 9 | 17.7 (±71.2) | 28 | 31.2 (±75.3) | - | - | 37 | 27.9 (±78.8) | 39 |
| Sulfadimethoxine | - | - | 6 | 27.9 (±50.5) | - | - | 6 | 27.9 (±50.5) | 31^*^ |
| Neomycin | 3 | 18.3 (±67.7) | 14 | 30.1 (±163.1) | - | - | 17 | 28.0 (±159.6) | 24 |
| Tilmicosin | 7 | 39.1 (±52.1) | 1 | 5.6 | - | - | 8 | 28.1 (±75.2) | 18 |
| Spectinomycin | - | - | 7 | 33 (±117.5) | - | - | 7 | 33.0 (±117.5) | 124 |
| Sulphamethoxazole | - | - | 6 | 32.8 (±97.2) | 1 | 33.8 | 7 | 33.0 (±88.1) | 27^*^ |
| Apramycin | - | - | 1 | 33.8 | - | - | 1 | 33.8 | 81 |
| Kitasamycin | - | - | 1 | 33.8 | - | - | 1 | 33.8 | - |
| Norfloxacin | 3 | 33.7 (±47.1) | - | - | - | - | 3 | 33.7 (±47.1) | - |
| Cefadroxil | 1 | 45 | - | - | - | - | 1 | 45.0 | - |
| Sulphathiazole | 1 | 45 | - | - | - | - | 1 | 45.0 | - |
| Florfenicol | 8 | 34.6 (±106.6) | 5 | 66.2 (±127.5) | - | - | 13 | 46.7 (±125.2) | 30 |
| Cephalexin | 1 | 67.5 | - | - | - | - | 1 | 67.5 | - |
| Sulfadimidine | - | - | 6 | 71.7 (±117.8) | - | - | 6 | 71.7 (±117.8) | 25^*^ |
| Methenamine | 1 | 320.6 | - | - | - | - | 1 | 320.6 | - |

*Including trimethoprim^**^ Including sulphonamide.
